# Evaluation of whole-genome sequencing of four Chinese crested dogs for variant detection using the ion proton system

**DOI:** 10.1186/s40575-015-0029-2

**Published:** 2015-10-08

**Authors:** Agnese Viluma, Shumaila Sayyab, Sofia Mikko, Göran Andersson, Tomas F. Bergström

**Affiliations:** Department of Animal Breeding and Genetics, Swedish University of Agricultural Sciences, Uppsala, Sweden

**Keywords:** Next-generation sequencing, Ion Proton, Whole-genome sequencing, Dog genome, Variant detection

## Abstract

**Background:**

Next generation sequencing (NGS) has traditionally been performed by large genome centers, but in recent years, the costs for whole-genome sequencing (WGS) have decreased substantially. With the introduction of smaller and less expensive “desktop” systems, NGS is now moving into the general laboratory. To evaluate the Ion Proton system for WGS we sequenced four Chinese Crested dogs and analyzed the data quality in terms of genome and exome coverage, the number of detected single nucleotide variants (SNVs) and insertions and deletions (INDELs) and the genotype concordance with the Illumina HD canine SNP array. For each of the four dogs, a 200 bp fragment library was constructed from genomic DNA and sequenced on two Ion PI chips per dog to reach mean coverage of 6–8x of the canine genome (genome size ≈ 2.4 Gb).

**Results:**

On average, each Ion PI chip yielded approximately 73.3 million reads with a mean read length of 130 bp (~9.5 Gb sequence data) of which 98.5 % could be aligned to the canine reference genome (CanFam3.1). By sequencing a single dog using one fragment library and two Ion PI chips, on average 80 % of the genome and 77 % exome was covered by at least four reads. After removing duplicate reads (20.7 %) the mean coverage across the whole genome was 6x. Using sequence data from all four individuals (four fragment libraries and eight Ion PI chips) the genome and exome coverage could be further increased to 97.2 and 94.3 %, respectively. We detected 4.83 million unique SNPs and 6.10 million unique INDEL positions across all individuals. A comparison between SNP genotypes detected with the WGS and the 170 K Illumina HD canine SNP array showed 90 % concordance.

**Conclusions:**

We have evaluated whole-genome sequencing on the Ion Proton system for genetic variant detection in four Chinese crested dogs. Even though INDEL calling with Ion Proton data is challenging due to specific platform errors, in case of SNP calling it can serve as an alternative to other next-generation sequencing platforms and SNP genotyping arrays, in studies aiming to identify causative mutations for rare monogenic diseases. In addition, we have identified new genetic variants of the Chinese Crested dog that will contribute to further whole-genome sequencing studies aimed to identify mutations associated with monogenic diseases with autosomal recessive inheritance.

**Electronic supplementary material:**

The online version of this article (doi:10.1186/s40575-015-0029-2) contains supplementary material, which is available to authorized users.

## Lay summary

Two different methods for sequencing DNA were developed independently in the 1970s by Fred Sanger and Walter Gilbert. The sequencing methods are generally referred to as “Maxam-Gilbert sequencing” and “Sanger sequencing”. For their discoveries that revolutionized genetic research, they were awarded the Nobel Prize in Chemistry in 1980. The Sanger sequencing method was used to sequence the human genome and the project was completed in 2003. The cost for the human genome project has been estimated to $3 billion and took more than ten years to accomplish. Shortly thereafter, several new sequencing methods became available allowing for more efficient sequencing of complete mammalian and plant genomes. The new methods relied on massively parallel sequencing and are referred to as next-generation sequencing (NGS) to reflect the technological leap from Sanger sequencing (first generation sequencing) and has truly led to a paradigm shift in biological research, resulting in a deepened understanding of complex biological systems. As a result of the recent accelerated development of NGS technologies, costs of whole genome sequencing have decreased dramatically and are approaching $1000 for sequencing of a complete mammalian genome.

With the introduction of smaller and less expensive “desktop” systems, NGS is now moving into the general laboratory. To evaluate the data quality obtained from one of the available desktop NGS-platforms called the Ion Proton system, we sequenced the genomes of four Chinese Crested dogs. The data quality was analyzed in terms of coverage of the dog genome (i.e. how many times each and every base of the dog genome was sequenced). In addition, the sequence data produced by the Ion Proton system for these four dogs, was compared to known genetic variants in public databases as well as to genetic variants detected in these four dogs using a genetic variant detection system not based on sequencing but rather by hybridization to known genetic variants found in dog genomes in general.

The results of our investigation showed that we obtained a sufficient coverage of the dog genome allowing us to find 90 % of all the genetic variants that was detected with the hybridization-based method. Thus, we conclude that the Ion Proton system can serve as an alternative to other NGS platforms in studies aiming to identify mutations associated with rare monogenic diseases. In addition, new genetic variants of the Chinese Crested dog were identified.

## Background

Next-generation sequencing (NGS) technology, both for whole-genome sequencing (WGS) and whole-exome sequencing (WES), has not only reduced the cost of sequencing individual genomes, but also provides a powerful and unbiased approach for large-scale detection of genetic variation [1], including single nucleotide polymorphisms (SNPs), insertion/deletions (INDELs) and copy number variations (CNVs). During the last decade different NGS platforms (e.g. Illumina HiSeq, Roche 454, SOLiD and PacBio) have been used to generate sequence data in specialized genome centers. In recent years, several “desktop” sequencing platforms such as Illumina MiSeq and NextSeq500, Ion Torrent PGM and Ion Proton have been introduced providing an alternative choice for WES and low coverage WGS of mammalian genomes.

In human studies, WES has successfully been used to discover mutations causing rare Mendelian disorders [2, 3] and also candidate mutations for complex disorders, e.g. mental retardation [4] and Charcot-Marie-Tooth neuropathy [5]. Recently, several canine disease mutations causing Imerslund-Grasbeck syndrome, disproportionate dwarfism, nasal parakeratosis, footpad hyperkeratosis, spinocerebellar ataxia and neuronal ceroid lipofuscinosis have been identified with combination of genome-wide association studies (GWAS) and WGS [6–10] and by WGS without prior GWAS [11, 12].

The first high quality draft of the canine reference genome sequence (*Canis familiaris*) was released in 2005 [13]. The most recent genome build (CanFam3.1) that covers 99.8 % of the euchromatic portion of the genome has an improved annotation incorporating RNA-Sequencing data from ten different canine tissues [14]. This together with publicly available genetic variation in dogs [15, 16] provides a solid resource for WGS analysis for discovering disease-causing mutations in dogs. According to the Online Mendelian Inheritance in Animals (OMIA) data base 256 Mendelian traits/disorders are registered in dog, of which approximately 30 % of the causative mutations remain unknown [17].

In this study we have evaluated the Ion Proton system, which uses semiconductor technology [18], for WGS of the canine genome in terms of genome and exome coverage, the number of detected variants (SNPs and INDELs) and the genotype concordance with Illumina HD canine SNP array.

## Results

### Whole-genome sequencing and alignment

**Fig. 1:**
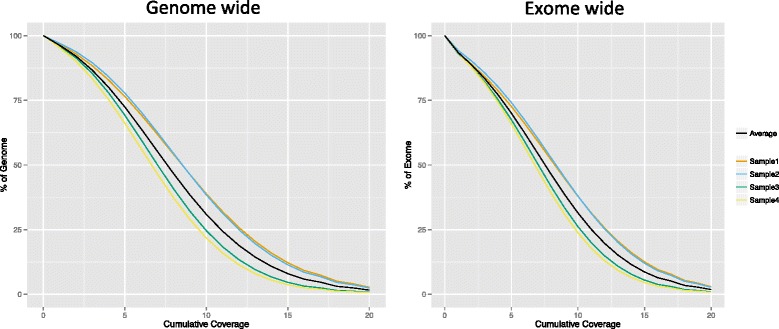
Cumulative base coverage distribution. The cumulative read depth analysis of raw binary alignment (BAM) files. Each dog sample sequenced from one library and two Ion Proton PI chips (9.5 Gb). The x-axis represents the minimum read depth per base and the y-axis the percentage of genome (*left panel*) and exome (*right panel*) that is covered

**Fig. 2:**
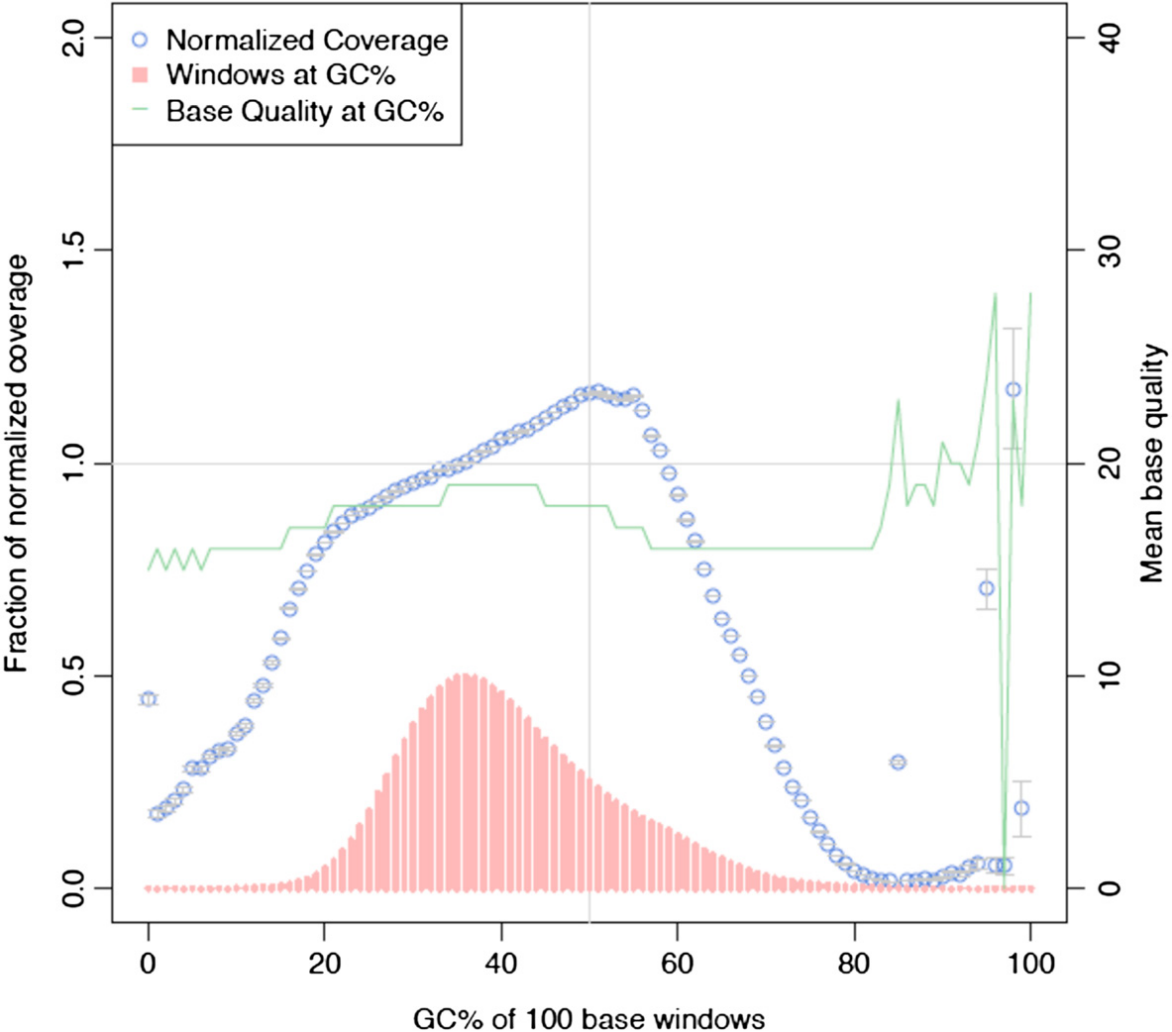
GC bias and the normalized coverage. Read coverage over the canine genome with respect to GC content, calculated in 100 bp windows (*in red color*) and in each window fraction of normalized coverage (*in blue color*) and plotted against the left y-axis. Mean base quality at GC % (*green*) is calculated and plotted against the right y-axis

Genomic DNA from four Chinese Crested dogs was sequenced on the Ion Proton system. For all four dogs, one 200 bp fragment library was constructed and sequenced on two Ion PI chips with 500 single nucleotide flows, ensuring that 200 bp read length can be achieved. The distribution of read lengths was similar on all eight chips with the highest peak around 150 bp (Additional file 1). On average, each chip produced 9.5 Gb sequence data, corresponding to ~73.3 million single reads with a mean read length of 130 bp (Additional file 2). The longest read reached 374 bp, but after approximately 160 bp an increased error rate was observed (Additional file 3). Sequence reads were mapped to the canine reference genome (CanFam3.1) using the TorrentSuite v.3.6.2. For all four dogs, on average 98.5 % of the reads could be aligned to the reference genome (Additional file 4), corresponding to a mean coverage of 8x per sample. Analysis of raw binary alignment map (BAM) files revealed that sequence reads on average covered 80 % of the whole genome and 77 % of exome with a read depth per base of four or greater (Fig. 1). After removal of duplicated reads (20.7 %), the average autosomal genome coverage was approximately 6x. Analyzing the read coverage over the canine genome with respect to GC-content (Fig. 2) showed expected coverage (a relative coverage of 1) where the GC-content was between 35 and 60 %. We detected a gradual drop in coverage if the GC content was less than 35 % or greater than 60 %. The mean base quality also deviated when the GC content was above 80 % as shown in Fig. 2.

### Variant detection

For each individual dog, using one sequencing library on two PI chips (Individual analysis) genetic variants were called with SAMtools, UnifiedGenotyper and HaplotypeCaller tool. This produced on average 2.4 million filtered SNVs and 0.7 million INDEL positions (Table 1) per dog and tool. The number of filtered INDELs called by SAMtools was more than three times higher than the number obtained by the UnifiedGenotyper tool and almost twice as high compared to the results from the HaplotypeCaller tool.

**Table 1:** Number of detected variants across different variant calling tools

	Combined analysis		Individual analysis^a^		
	SAMtools	UG	SAMtools	UG	HC
	Nr of SNVs (Ti/Tv^b^)	Nr of SNVs (Ti/Tv^b^)	Nr of SNVs (Ti/Tv^b^)	Nr of SNVs (Ti/Tv^b^)	Nr of SNVs (Ti/Tv^b^)
Total	5 165 528 (2.03)	4 802 404 (1.93)	3 065 136 (2.12)	2 650 589 (2.04)	2 410 162 (2.18)
Filtered	4 255 671 (2.19)	4 471 459 (2.01)	2 280 929 (2.22)	2 525 133 (2.09)	2 363 010 (2.19)
Known^a^	1 423 628 (2.39)	1 374 703 (2.40)	860 320 (2.44)	896 616 (2.42)	873 093 (2.44)
Novel	2 832 043 (2.10)	3 096 756 (1.87)	1 420 609 (2.11)	1 628 517 (1.94)	1 489 918 (2.06)
	Nr of INDELs	Nr of INDELs	Nr of INDELs	Nr of INDELs	Nr of INDELs
Total	11 750 679	3 539 988	3 778 222	341 366	1 295 497
Filtered	5 635 914	2 764 772	1 157 392	334 763	644 610
Known^c^	4 188	4 129	1 493	1 001	1 422
Novel	5 631 726	2 760 643	1 155 899	333 761	643 188

^a^Average result from four individuals; ^b^Transition-Transversion ratio; ^c^Known variants in dog [16], *UG* UnifiedGenotyper tool, *HC* HaplotypeCaller tool

**Fig. 3:**
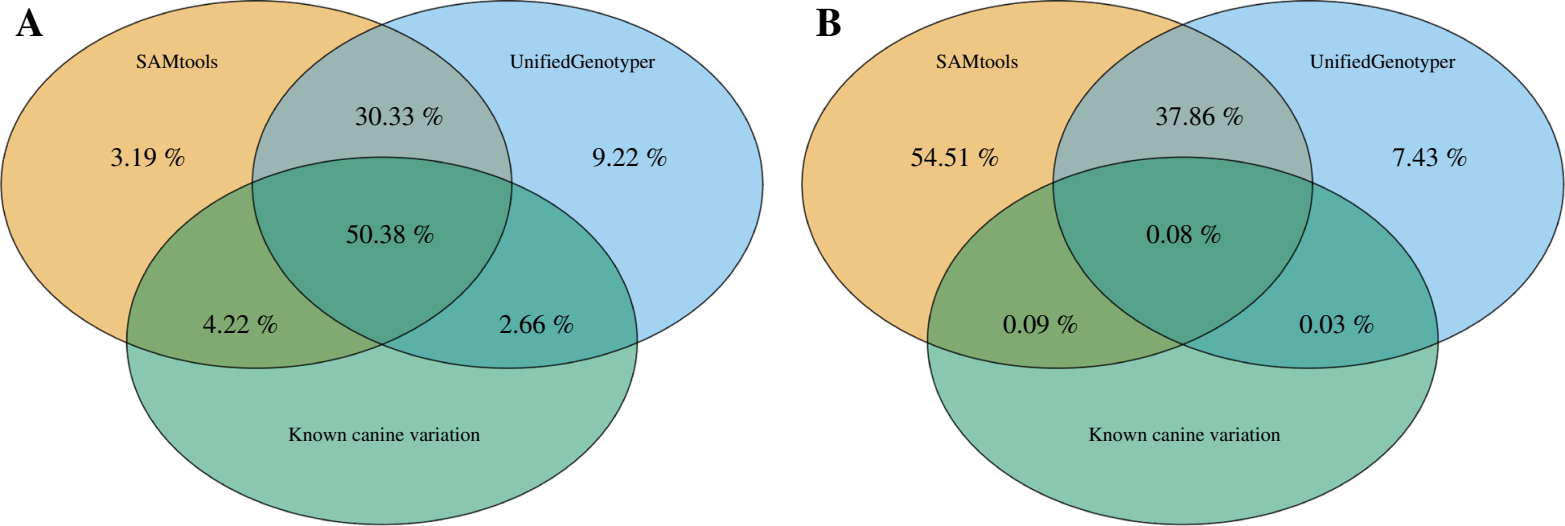
Detected variant overlap with already known genetic variation in dogs for SNVs and INDELs. Comparison of two different variant calling tools (SAMtools and UnifiedGenotyper) showing overlap between detected (**a**) SNVs and (**b**) INDELs (*yellow circle* for SAMtools; *blue circle* for UnifiedGenotyper) with already known variants in dogs (*green circle*). The proportion of a particular overlap category is shown in percentage of the total unique SNV (4.83 million) or INDEL (6.10 million) number detected by both tools. Non-overlapping parts of both tools represent variants detected only with a particular tool

By combining sequence data from all eight Ion PI chips (Combined analysis), genetic variants (SNVs and short INDELs) were called using SAMtools and UnifiedGenotyper (Table 1). From those, ~80 % of SNV and ~38 % of INDEL calls were identified with both tools (Fig. 3). Merging of the variants from both tools resulted in 4.83 million SNVs and 6.10 million INDEL positions, which fulfilled filtering conditions. From those, ~57 % of SNV and ~0.2 % of INDEL positions were concordant with positions of known variation in the canine genome.

### Concordance with Illumina HD Canine SNP array

**Fig. 4:**
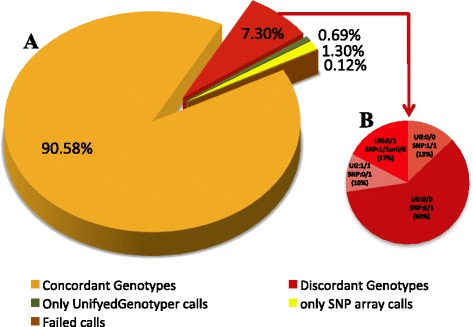
Concordance with Illumina HD canine SNP array. **a** Left pie-chart: Concordance between genotypes from two individual dogs detected by the 170 k Illumina HD canine SNP array (CanineHD BeadChip) and called variants from NGS data using UnifiedGenotyper. 90.58 % of the genotypes were concordant, 7.30 % were discordant, 0.69 % were only called by UnifiedGenotyper, 1.30 % were called only by the SNP array and 0.12 % failed with both UnifiedGenotyper and SNP array. **b** Right pie-chart: Distribution of discordant genotypes based on the type of discordance. The reference allele is coded with 0 and alternative allele with 1. Thus 60 % of discordant genotypes were called homozygous for reference allele with UnifiedGenotyper and heterozygous with the SNP array (0/0:0/1); 17 % (0/1:1/1 or 0/0); 13 % (0/0:1/1); and 10 % (1/1:0/1)

Two of the sequenced individuals were also genotyped with the 170 K CanineHD BeadChip (Illumina) comprising 174 037 markers. On average, more than 90 % of the SNVs were concordant with the SNP array data, but in 7.3 % of called genotypes, discordance was observed (Fig. 4). The most common mismatch, that constituted 60 % of discordant genotypes, was observed when the individual had been called as homozygous for the reference allele by UnifiedGenotyper, but heterozygous by SNP array (Fig. 4). The average read depth in discordant calls was 5x with a mean SNP Phred quality score of 19 while the mean Phred quality score of all 174 037 called positions was 90.

### Library merging simulation

**Fig. 5:**
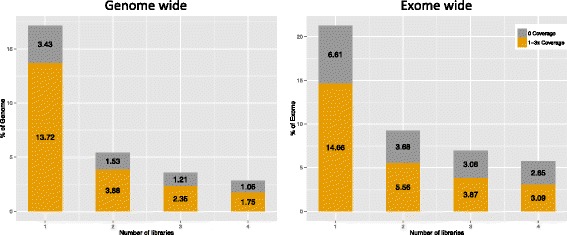
Proportion of genome- and exome-wide coverage by merging reads from different libraries. Left panel: The proportion of the genome with zero coverage (*gray color*) or with 1–3x coverage (*orange color)* when one library was compared with merging the reads from two, three or four sequencing libraries. With one library sequenced with two Ion Proton PI chips 3.43 % of the genome was not covered (0 coverage) and 13.72 % had coverage of 1–3x (considered as low coverage). With merging of the reads from four libraries 1.06 % had zero and 1.75 % had 1–3x coverage. Right panel: Similarly, the proportion of the exome with zero coverage (gray color) or with 1–3x coverage (*orange color)* is shown

To evaluate the potential increase of genome coverage when using more libraries and chips, a library merging simulation was performed (Fig. 5). In comparison to using one library and two PI chips, combining sequence data from two different libraries (Additional file 4) increased the covered proportion of the genome with four or more reads from 82.9 % (Sample 1) to 94.6 % (Samples 1 and 2). Similarly, the exome-wide coverage increased from 79.2 % (Sample 1) to 90.8 % (Samples 1 and 2). By merging all four libraries, this proportion could be further increased to 97.2 and 94.3 %, respectively. We analyzed regions in the canine reference genome that did not contain any aligned reads in our data set, in total, 24.8 Mb of the genome and 1.4 Mb of the exome. These regions were characterized in terms of gaps in the reference genome, repeats, CpG Islands (Table 2) and GC content (Fig. 2). After eliminating bases that were not covered due to gaps (all positions represented by N in the reference sequence) we were left with 14.8 Mb of genome and 1.3 Mb of exome lacking coverage. Large proportion of these bases, ~33 % genome-wide and ~73 % exome-wide, was located in known repeat regions as defined by RepeatMasker. Smaller proportion of non-covered bases, ~30 % genome-wide and ~19 % exome-wide, overlapped with positions of known CpG Islands. The average GC content of regions with low coverage and those lacking coverage in exonic regions were significantly (*p* < 2.2 × 10^−16^) higher than the average GC content of the whole exome.

**Table 2:** Characterization of regions not covered after merging all four libraries

Features	Genome-wide	Exome-wide
Bases not covered	24 811 567	1 384 635
N bases in reference sequence (gaps)	10 040 013	42 019
Total not covered bases excluding gaps	14 771 554 (0.63 % of genome)	1 342 616 (2.57 % of exome)
Bases not covered in repeat regions	4 912 354	983 511
Bases not covered in CpG Islands	4 375 887	253 643

## Discussion

Whole-genome sequencing of four Chinese Crested dogs was performed to evaluate the Ion Proton system with regard to the genome and exome coverage, the number of detected variants (SNVs and INDELs) and the genotype concordance with Illumina HD canine SNP array. With one fragment library and two PI chips per individual an average of 19 Gb sequence data was produced allowing to cover 80 % of the genome and 77 % of the exome with at least four reads. In principle, a variant position can be called with a single read, but with an increased rate of false positives and missed alternative alleles. Depth of coverage is one of the most important caveats of variant calling from NGS data. It has been shown that both sensitivity and specificity of the variant calling based on low coverage sequencing can be improved by adding more individuals to the analysis [19, 20]. In this study of Chinese crested dogs, we detected on average 3.5 million variants (2.4 million SNVs and 1.2 million INDELs) for each dog with individual variant calling approach. In other WGS studies of dogs with a higher mean read coverage (20–34x), the number of reported variants (SNVs and INDELs) observed in single dogs from other breeds ranged between 6.1 and 7.4 million [10–12]. This indicates that a fourfold increase of mean read coverage in our study could have resulted in at least a doubling of called variants. In our combined variant calling across all four Chinese Crested dogs and two variant calling tools, we detected 10.9 million unique variants (4.8 million SNVs and 6.1 million INDELs). In a study where five pools of domestic dogs and one pool of 12 wolfs were sequenced with low-coverage (~6x) on the AB SOLiD system resulted in the discovery of 3.7 and 3.8 million SNV positions in the wolf and dog pools, respectively [15].

We observed a high concordance (>80 %) among the two variant calling tools for SNV detection and more than a half of detected variants were overlapping with known SNP variation in Ensembl (Variation Release 77), providing a confident dataset of known and novel variation of the dog genome. However, the number of detected INDEL variants should be interpreted with caution since only 0.2 % of the detected INDELs overlapped with Ensembl data. We interpret this discrepancy to be a result of the two types of error, flow-call accuracy and high-frequency INDEL errors, described for the IonTorrent Personal Genome Machine (PGM) semiconductor platform [21]. Furthermore, we obtained high variation between the two different variant calling tools, GATK and SAMtools, which may be explained by differences of data preprocessing applied before variant calling (local realignment around the possible INDEL positions and base quality score recalibration). It is likely that the low coverage was substantially contributing to the large differences observed between the tools. Thus, with a higher coverage it is possible that the difference would be less conspicuous. Lastly, only limited information to estimate and eliminate false positives of INDELs is currently available.

As genotype quality control, we used Illumina HD array results from two of our four sequenced individuals and the concordance detecting the correct genotype was found to be over 90 %. The most common inconsistency was the inability of the sequencing data to detect positions that were genotyped as heterozygous by the Illumina HD array. The average mean read depth at these positions was 5x, illustrating the obvious risk for missing genetic variants using a low coverage sequencing approach. This is a good indication for the quality depth filtering threshold to increase genotype quality, keeping in mind that all alleles might not be observed even at 10x coverage [22] and in cases of low-coverage sequencing would eliminate considerable part of true positives, as for example in our study, more than 25 % of genome has ≤ 5x coverage.

To evaluate the possible improvement of mean read depth coverage and proportion of covered genome/exome by increasing fragment library and PI chip number per individual we performed a library merging simulation. This analysis showed that the most substantial decrease in not covered and low covered areas of the genome was when adding a second sequencing library. The addition of a third and a fourth library, only slightly decreased the proportion of not covered and low covered regions. This suggests that two libraries per individual, sequenced on at least four PI chips is an optimal design balancing cost and coverage outcome with the IonProton system.

After merging all four available libraries, around 1 % of genome and almost 3 % of exome had no coverage. Analysis of those regions suggests that most of the not covered exome locations correspond to the known repeat region coordinates and alignment algorithms could be adjusted to address this issue. A considerable part of those regions was also overlapping with CpG islands, which is a common pitfall for the PCR-based sequencing platforms [23]. Thus, there is a high risk of missing causative variants located in the promoter and exonic regions with high GC content. For example, a recessive mutation causing Powderpuff genotype in Chinese Crested dogs could not be detected in our data set, due to lack of coverage in exon 1 of the *FOXI3* gene, which is in fact a repetitive GC rich exon. After eliminating these most common issues causing lack of coverage there was still a small fraction of non-covered bases that could not be explained.

Most of the previously reported dog genome re-sequencing studies have used WGS approach, but with the development of improved targeted WES enrichment kit (total size 52,9 Mb) for the dog based on CanFam3.1 identification of causative mutations by exome capturing may become an important alternative [24]. Theoretically, one Ion Proton PI chip would be sufficient to generate 180x coverage of the enriched exome. However, WGS has the advantage over WES as the annotation of the canine genome is constantly being improved, and importantly, it also enables detection of causative mutations located in noncoding regions such as promoters, enhancers, lncRNAs, miRNAs and ultra-conserved elements [25].

## Conclusions

We have evaluated Ion Proton system for genetic variant detection in whole-genome sequences from four individual dogs. Number of reads generated per individual library on two PI chips was sufficient to cover about 80 % of genome and 77 % exome at least four times and allowed detection of 2.4 million SNV positions with 90 % chance of correct genotype. A better result and decrease in false positive calls can be achieved by increasing library and chip number per individual or using combined analysis for variant calling that in our case resulted in 4.83 million unique SNV and 6.10 million INDEL positions. Even though INDEL calling with Ion Proton data is challenging due to specific platform errors, in case of SNP calling it can serve as an alternative to other next-generation sequencing platforms and SNP genotyping arrays. This approach can contribute to further whole-genome sequencing studies aimed to identify causative mutations of monogenic diseases with autosomal recessive inheritance. In addition, we have contributed new genetic variants of the Chinese Crested dog.

## Methods

### Sampling and alignment

Genomic DNA from four Chinese Crested dogs was extracted from peripheral blood leukocytes, using 1 ml blood on a QIAsymphony SP instrument and the QIAsymphony DSP DNA Kit (Qiagen, Hilden, Germany). One microgram of genomic DNA was fragmented using the Covaris S2 instrument (Covaris, Inc. Woburn, MA) and library preparation was performed using the Ion Xpress™ Plus Fragment Library Kit for AB Library Builder™ System followed by five cycles of amplification. Emulsion PCR was done on the Ion OneTouch™ 2 system with Ion PI™ Template OT2 200 Kit v2 chemistry (Life Technologies, Thermo Fisher Scientific, Waltham, MA). Enrichment was conducted using the Ion OneTouch™ ES (Life Technologies). Samples were loaded on two Ion PI™ chips Kit v2 and sequenced on the Ion Proton™ System using Ion PI™ Sequencing 200 Kit v2 chemistry (200 bp read length, Life Technologies).

Reads were aligned to the canine reference genome sequence (CanFam3.1.) using TorrentSuit 3.6 software with default settings. We further assessed the quality of obtained alignments using standalone versions of FastQC v0.7.2 [26].

### Coverage analysis

Analysis of the coverage distribution of individual raw binary alignment map (BAM) files and files from library merging simulation were performed with Genome Analysis Tool Kit (GATK) v.2.7 [27] PerBaseDistribution tool. Calculation of the mean read depth and coverage distribution visualization was done with RStudio v.0.97.551 [28]. The cumulative distribution describing the number of reads per base (depth) was analyzed at two levels: genome- and exome-wide using Ensembl transcripts, downloaded from UCSC Genome Browser [29, 30]. Regions with no coverage and low coverage regions (up to three reads) were extracted by GATK tool CallableLoci and represented both, genome- and exome-wide.

### GC-bias

To estimate the GC-bias for each sample we used CollectGcBiasMetrics in Picard tools v.1.69. [31]. For each 100 bp window the GC content was calculated over the reference sequence. To assess the GC-bias with respect to coverage, the ratio of coverage in each bin versus the mean coverage of all GC bins were plotted with mean base quality.

### Library merging simulation

In order to evaluate to what extent the increase of depth and libraries per sample would improve the coverage of the genome/exome we performed a library merging simulation, assuming that all four present libraries could represent four different libraries created from one sample. Library merging simulation was done by stepwise merging of raw BAM files with SAMtools v.0.1.19 merge function [32] which resulted in three additional merged BAM files of two, three and four libraries. The procedure was done in a stepwise manner starting with the library having the highest number of reads and consecutively adding libraries with decreasing number of reads. The genomic coordinates of regions that remained not covered after merging all four libraries were extracted and intersected using BEDtools software suite v2.16.2 [33] with available features like reference gaps, repeats and CpG Islands extracted with the UCSC Table Browser data retrieval tool [34, 35]. To compare GC content of all Ensembl genes and low/no coverage regions after merging all four libraries a pairwise *t*-test was used.

### Preprocessing alignment and variant calling

Alignment preprocessing steps and variant calling was done following GATK Best Practices guidelines [36]. For each raw BAM file we marked and removed the duplicate reads with Picard (v.1.69) using the tool MarkDuplicates. Next, we applied GATK duplicate removal, base quality score recalibration, INDEL realignment, variant calling and filtration using standard hard filtering parameters [19, 36]. For detection of SNVs and INDELs, we applied the GATK tools UnifiedGenotyper and HaplotypeCaller (due to the computational demands was only available for individual analysis), as well as the bcftools utility in SAMtools for variant discovery [32]. Publically available genetic variation (SNPs and INDELs) in the canine genome [16] were used as “true positives” in base quality score recalibration and variant calling with UnifiedGenotyper and HaplotypeCaller. The variant calling was done across all four samples simultaneously (combined analysis) as well as separately on each individual (individual analysis).

### Variant comparison

To evaluate the concordance among variant calling softwares, variant call format (VCF) file comparisons were done using both the combined and individual analysis described above. All VCF comparisons were done with vcf-compare in VCFtools v.0.1.8a [37] and visualized with R package VennDiagram [38].

### Concordance with Illumina HD Canine SNP array

Two of the sequenced individuals were genotyped with the 170 K Illumina HD canine SNP array (CanineHD BeadChip) with on average more than 70 markers per Mb. The concordance between the SNP array genotyping and the SNVs identified by NGS in the respective samples was done using GenABEL v.1.7–6 [39], custom perl scripts and BEDtools v. 2.16.2 [32]. We first converted the marker positions of the SNP array in CanFam.2.0 to BED format. To map the positions from CanFam 2.0 to CanFam3.1, we used the liftover tool [40]. For each position, the reference allele was extracted from the CanFam3.1 reference assembly. A custom perl script was used to check the accuracy of SNP array overlap with the SNVs called by UnifiedGenotyper.

### Availability of supporting data

The data sets (four BAM files and one VCF file) supporting the results of this article are available in the European Nucleotide Archive (ENA) repository, [study accession number: PRJEB10523, http://www.ebi.ac.uk/ena/data/view/PRJEB10523].

## Ethics approval and consent to participate

All samples were obtained with informed dog owner consent. Ethical approval was granted by the Swedish Animal Ethical Committee Dnr C12/15.

## Additional files

Additional file 1:
**Read length distribution.** The histograms show the read length distribution of each Ion PI™ chip. For each of the four dogs, one genomic library was constructed and sequenced on two Ion PI™ chips. (PDF 575 kb) Additional file 2:
**PI chip productivity and per base quality scores.** Table describing the yield and base quality of eight sequencing chips. (XLSX 40 kb) Additional file 3:
**Error per cycle.** Graph describing the error rate per each cycle that corresponds to the number of base pairs in each read. Different colors represent results from 8 PI chips. (PDF 17 kb) Additional file 4:
**Alignment statistics.** Table describing the alignment statistics (percentage of mapped and duplicate reads). (XLSX 42 kb)
