# Supplementary figures and images for: Evaluation of whole-genome sequencing of four Chinese crested dogs for variant detection using the ion proton system
Source: Canine Genet Epidemiol. 2015 Oct 8;2:16. doi: 10.1186/s40575-015-0029-2 (PMC4599337; doi:10.1186/s40575-015-0029-2)

# Additional file 1 - Read length distribution

Sample 1

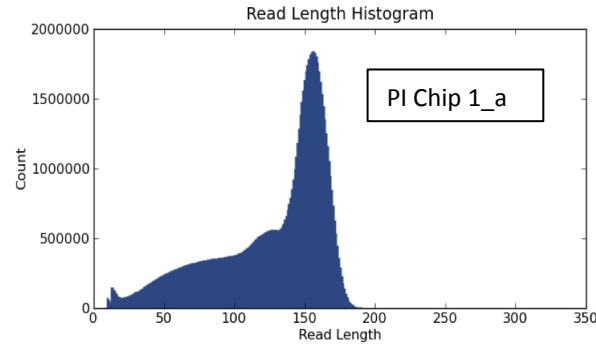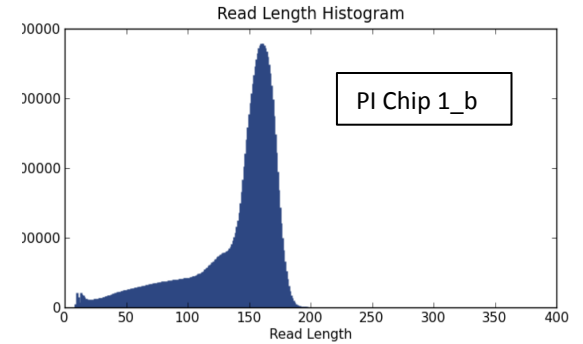

Sample 2

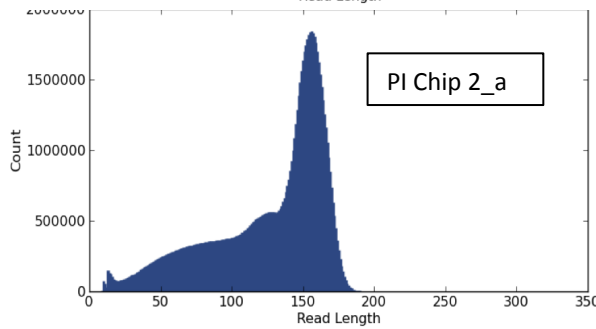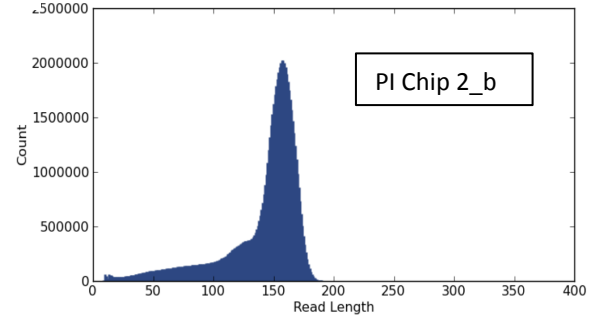

Sample 3

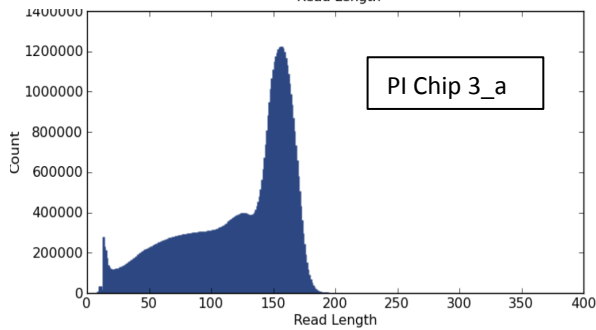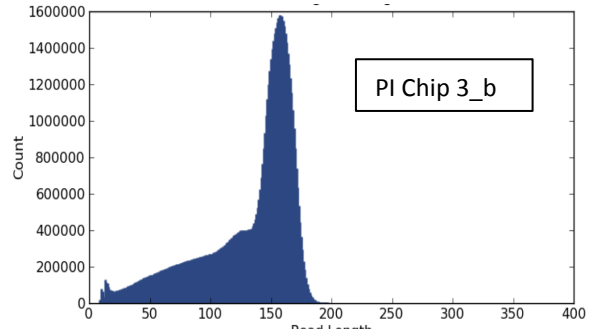

Sample 4

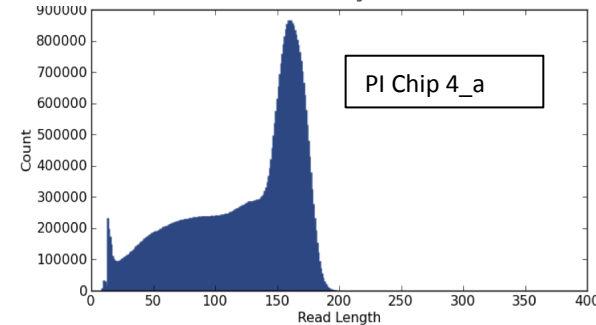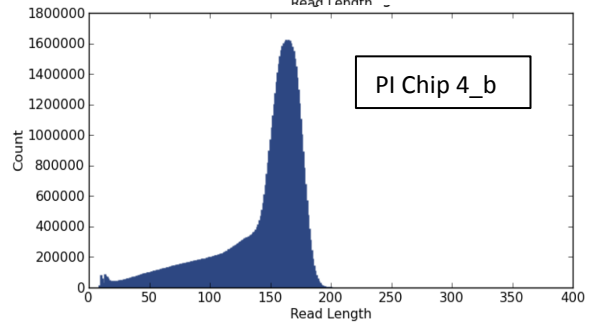

Supplement: Additional file 1: — Read length distribution. The histograms show the read length distribution of each Ion PI™ chip. For each of the four dogs, one genomic library was constructed and sequenced on two Ion PI™ chips. (PDF 575 kb) [file 40575_2015_29_MOESM1_ESM.pdf]

Error Per Cycle all readgroups

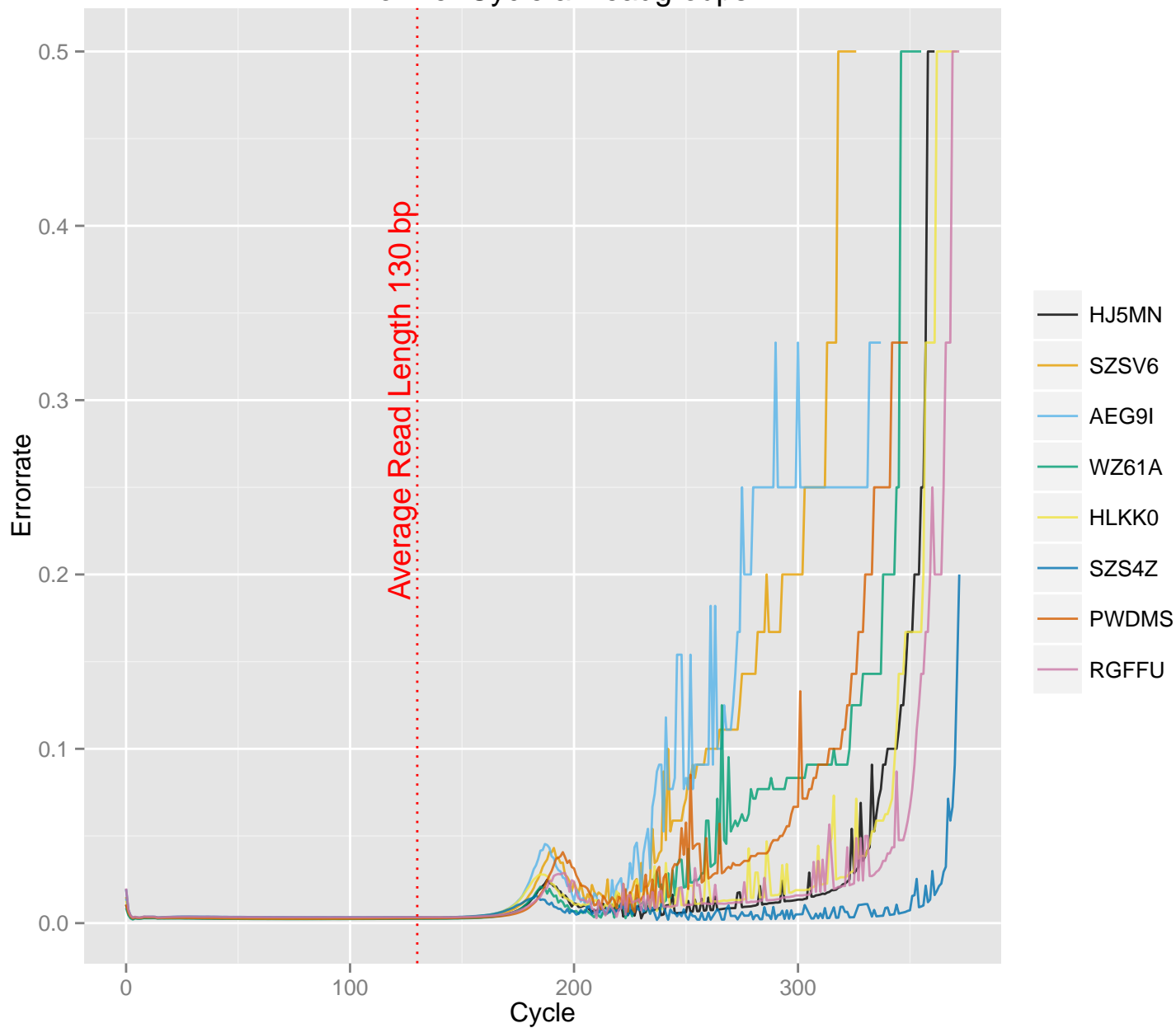

Supplement: Additional file 3: — Error per cycle. Graph describing the error rate per each cycle that corresponds to the number of base pairs in each read. Different colors represent results from 8 PI chips. (PDF 17 kb) [file 40575_2015_29_MOESM3_ESM.pdf]
